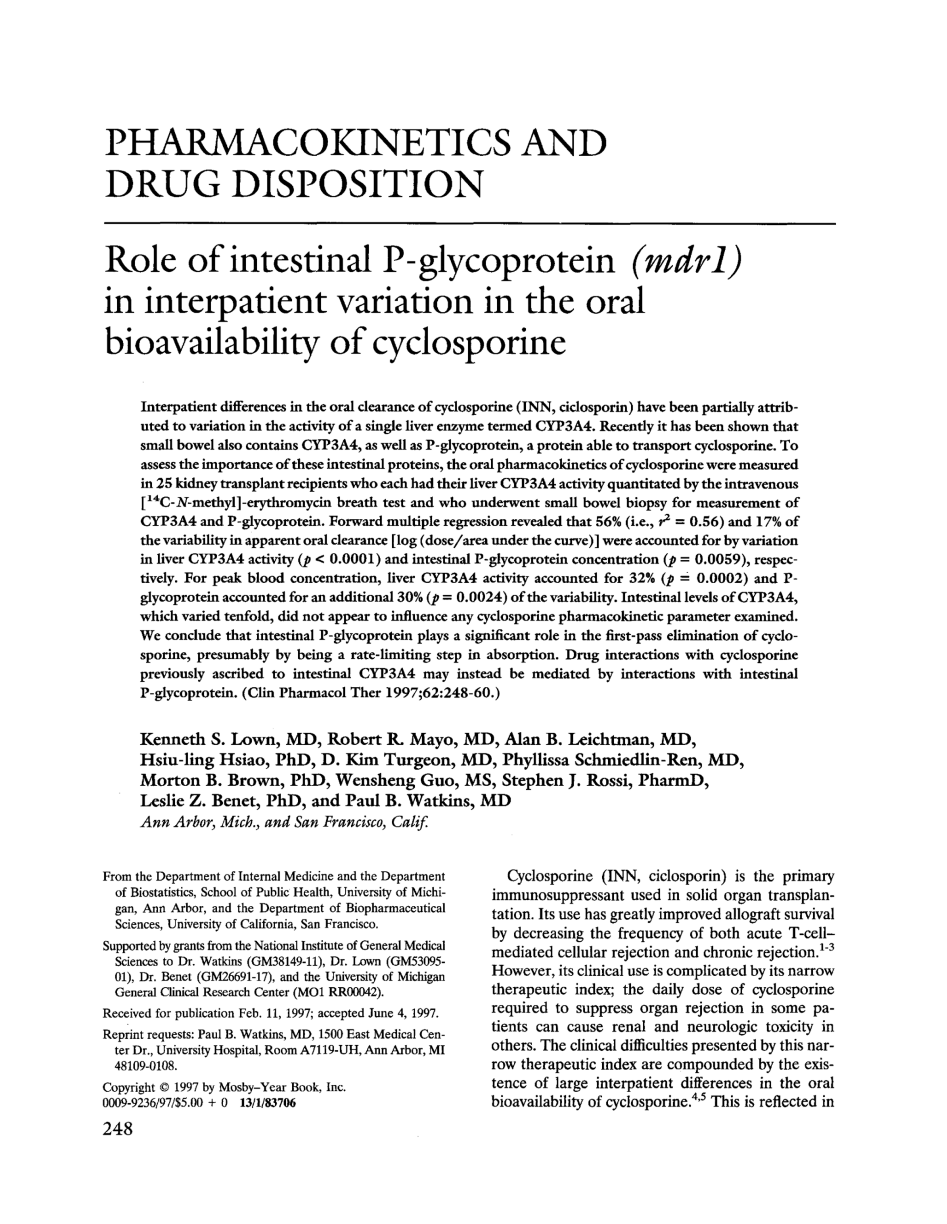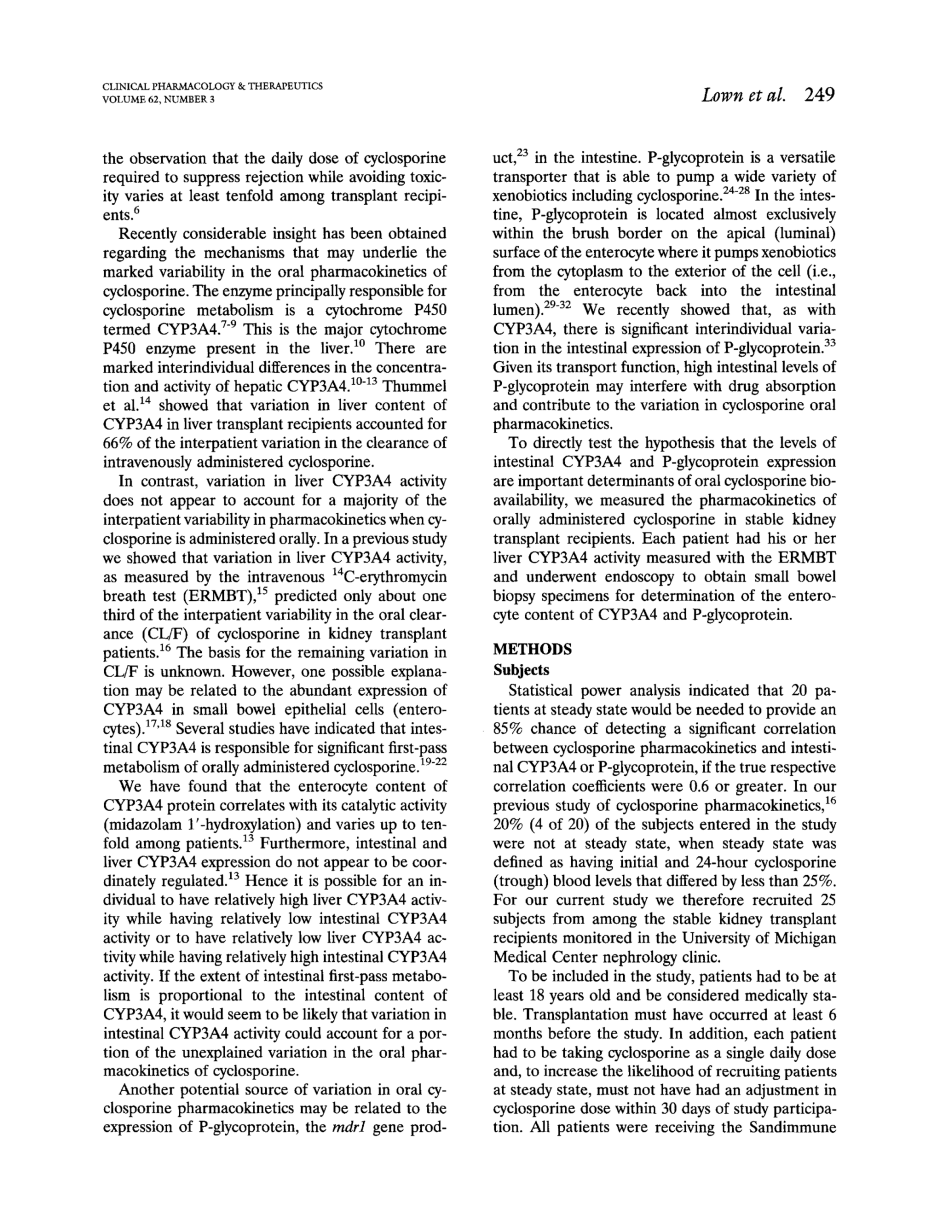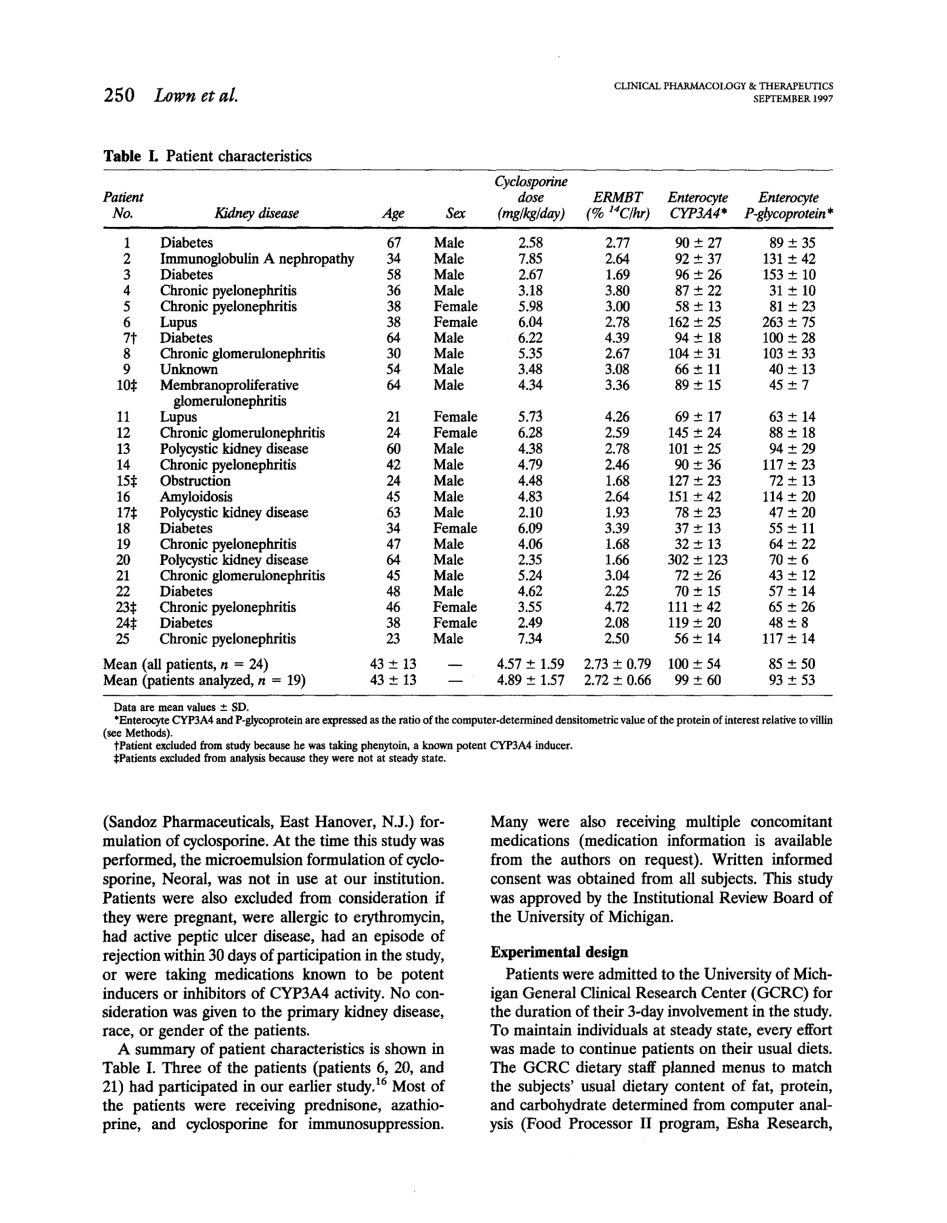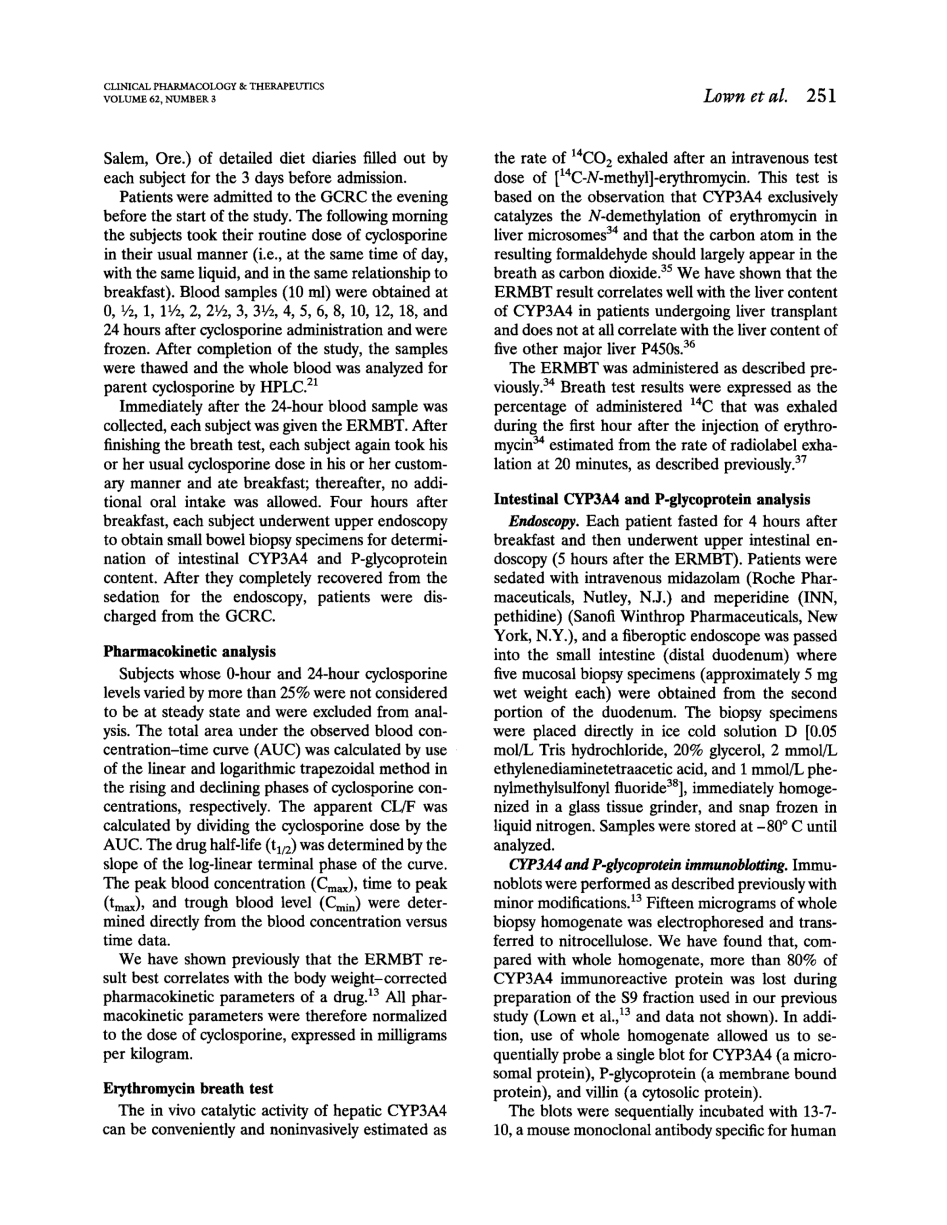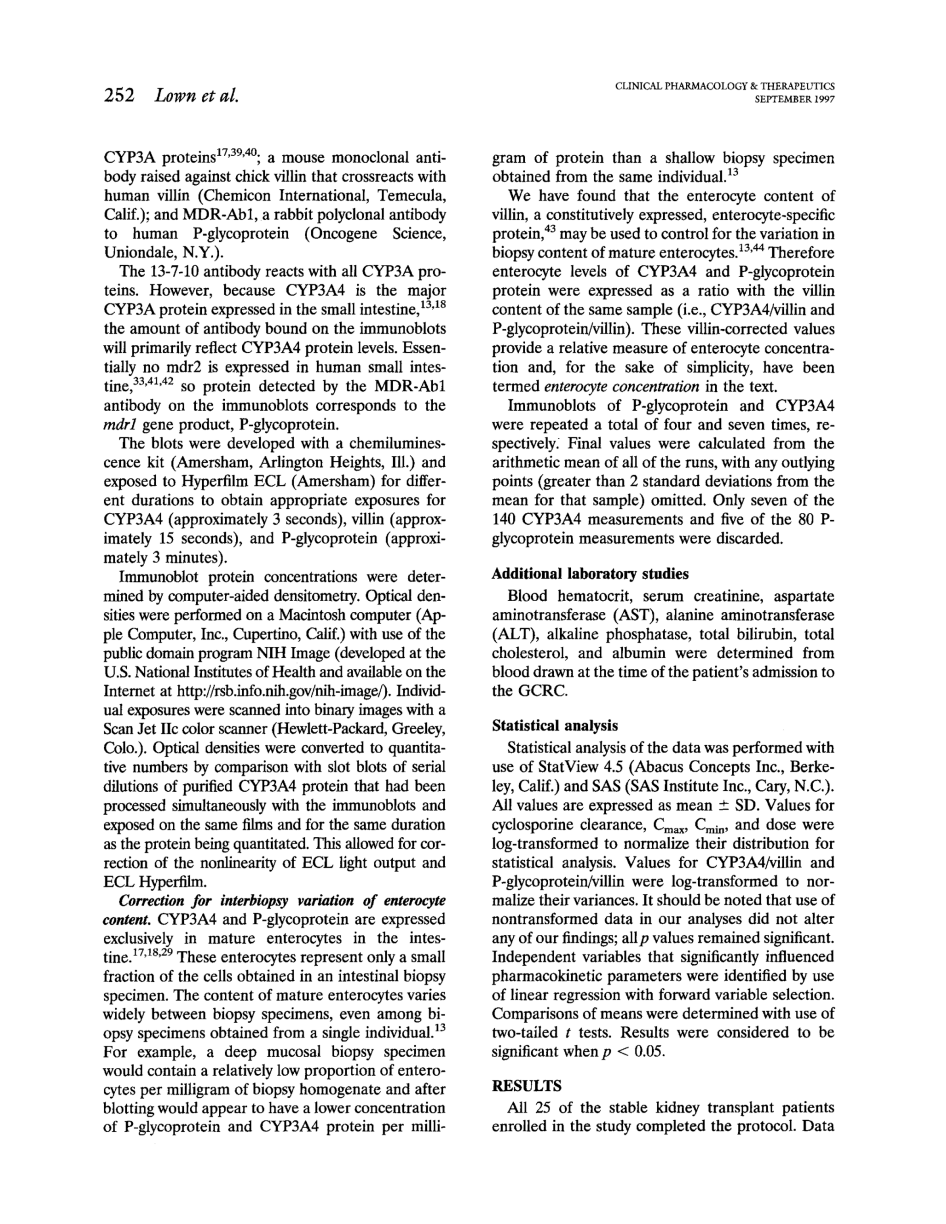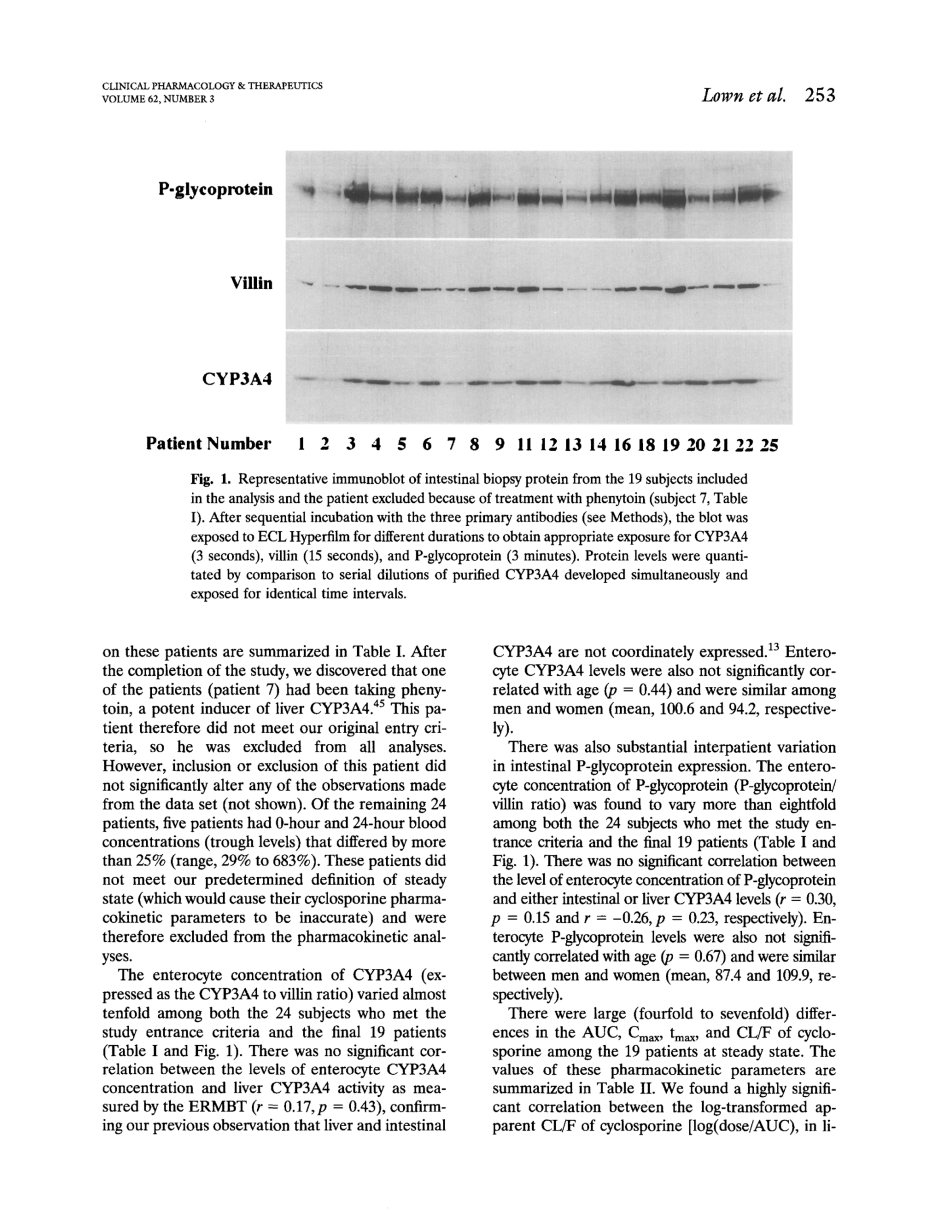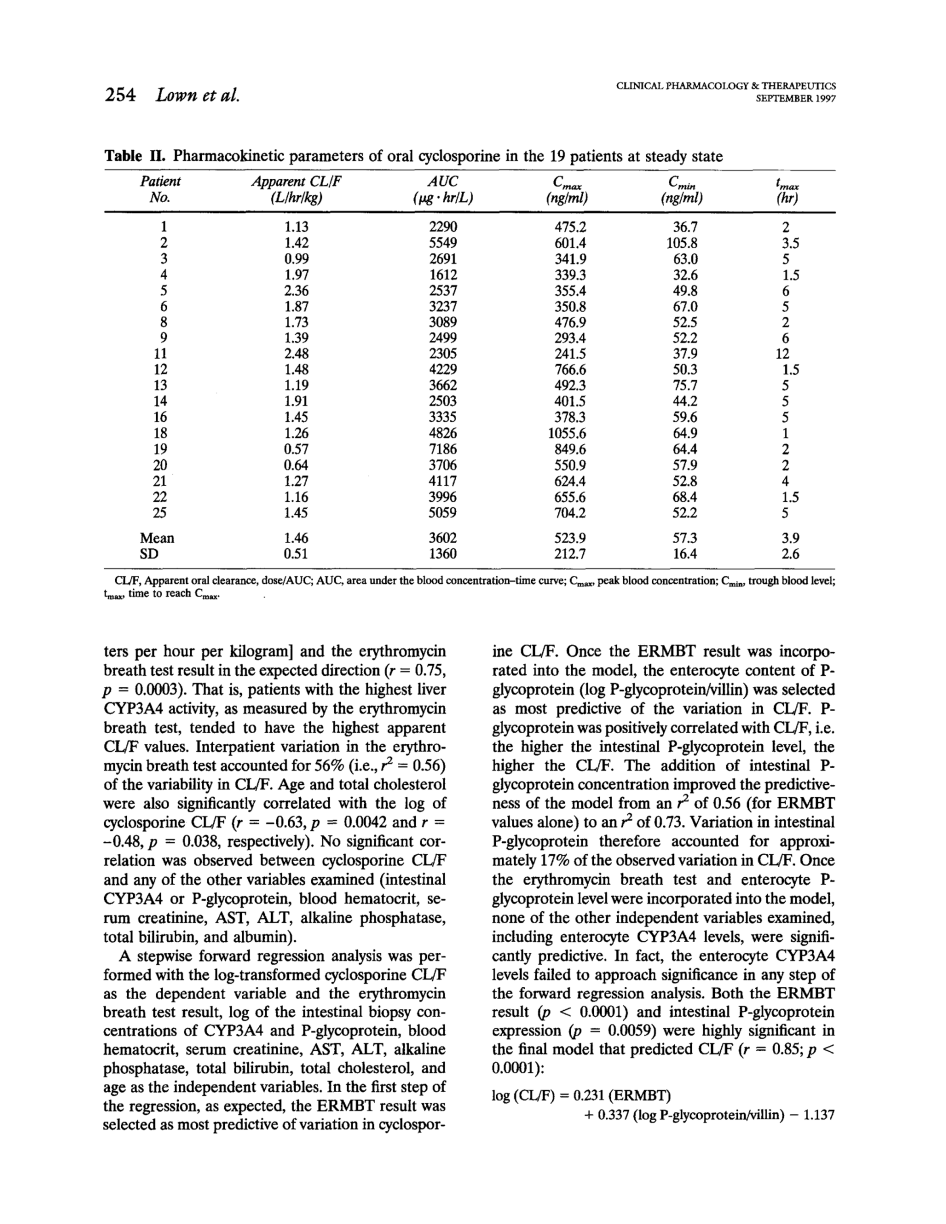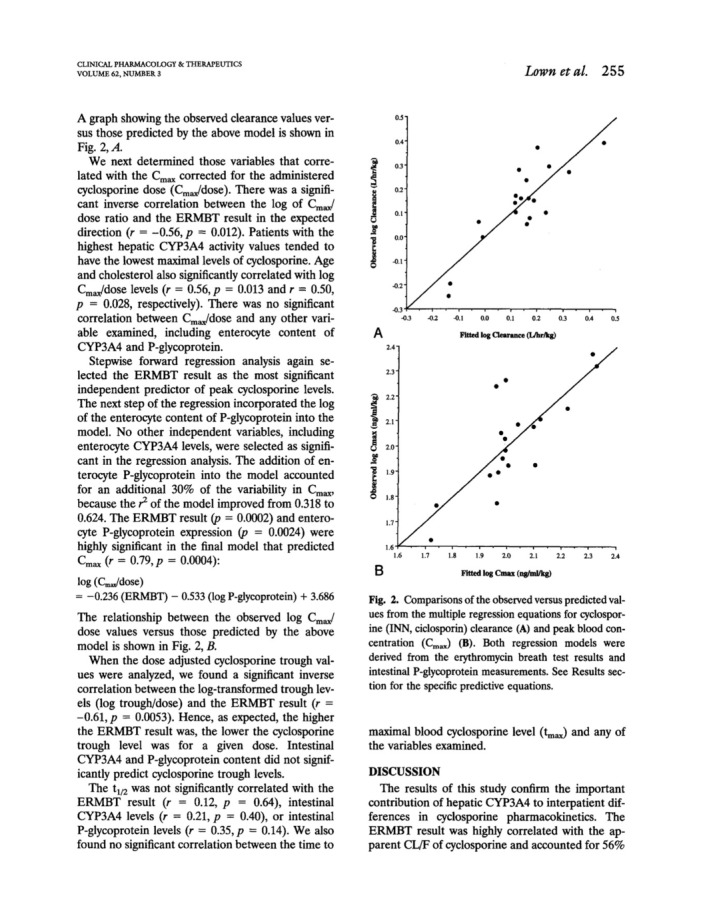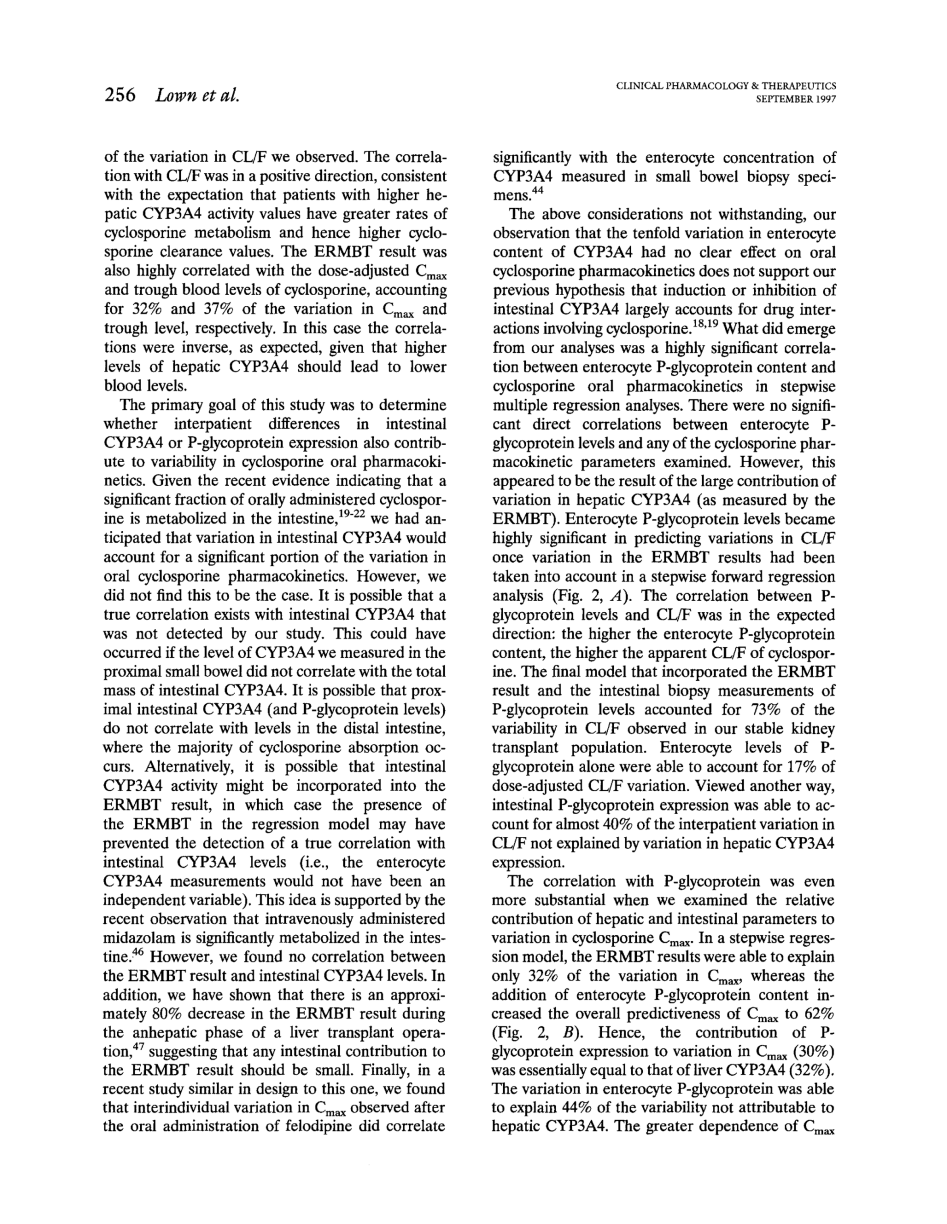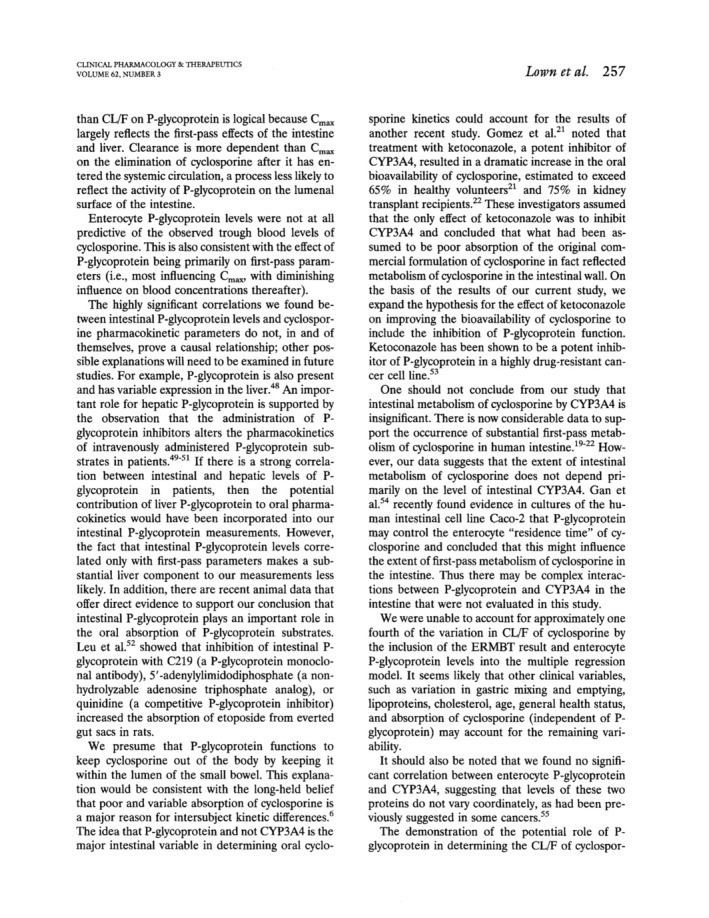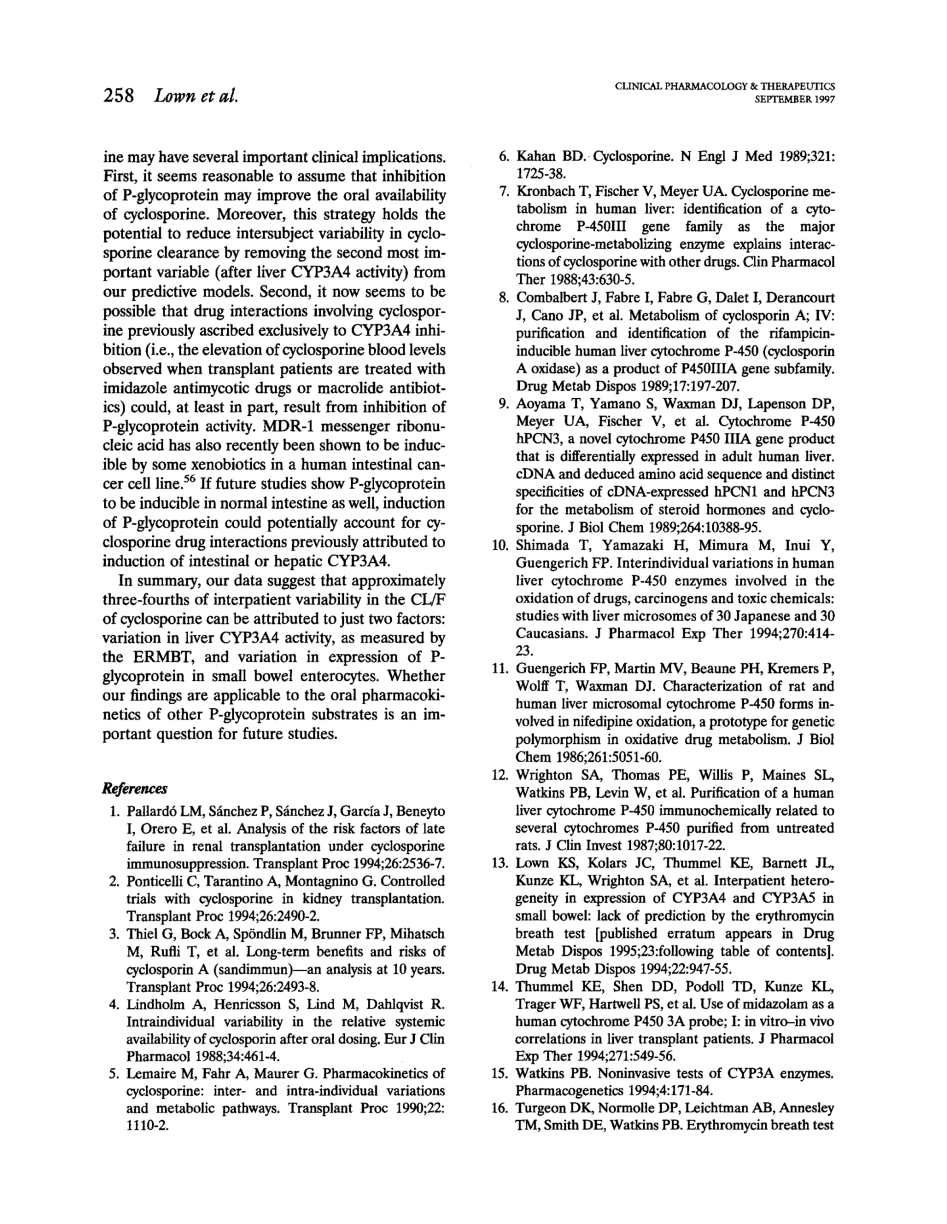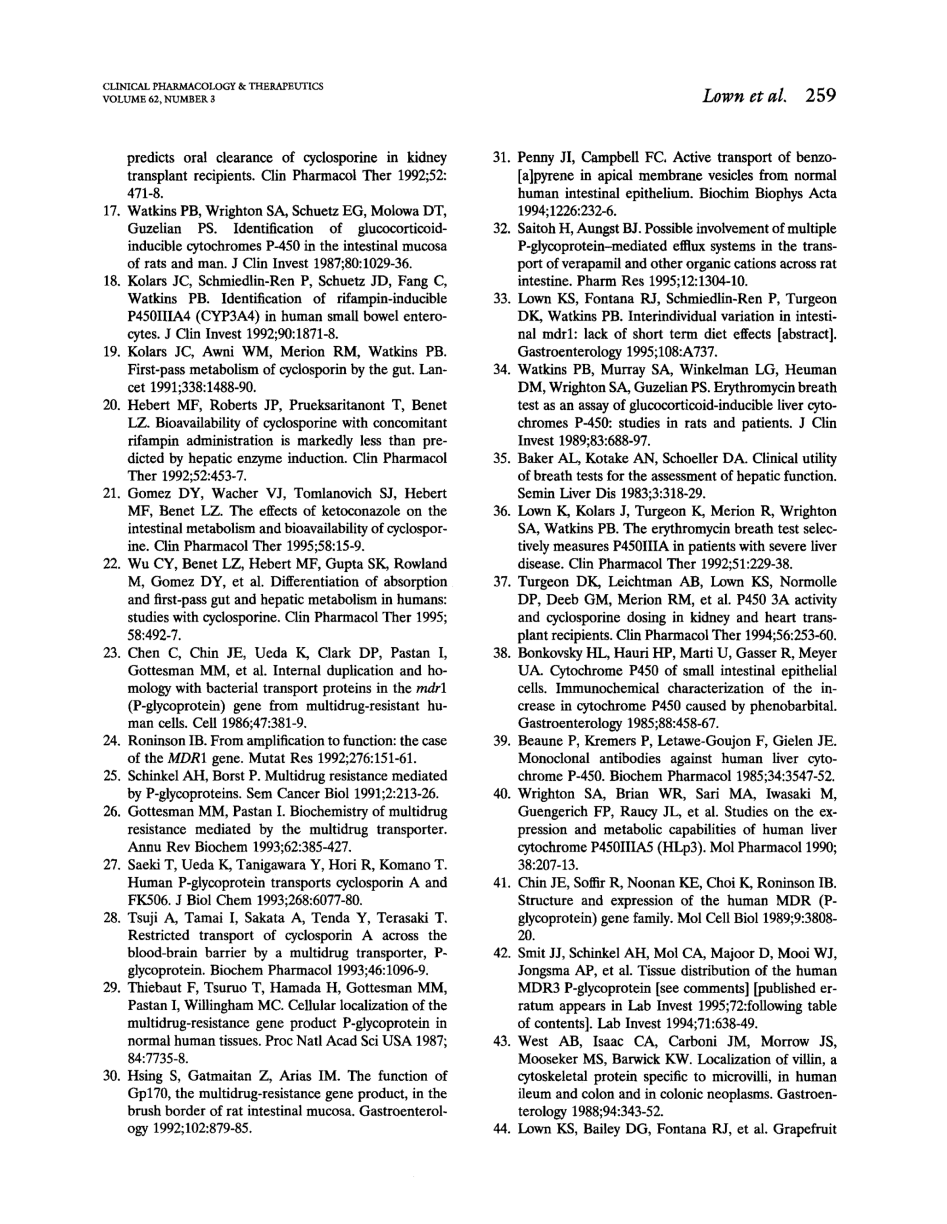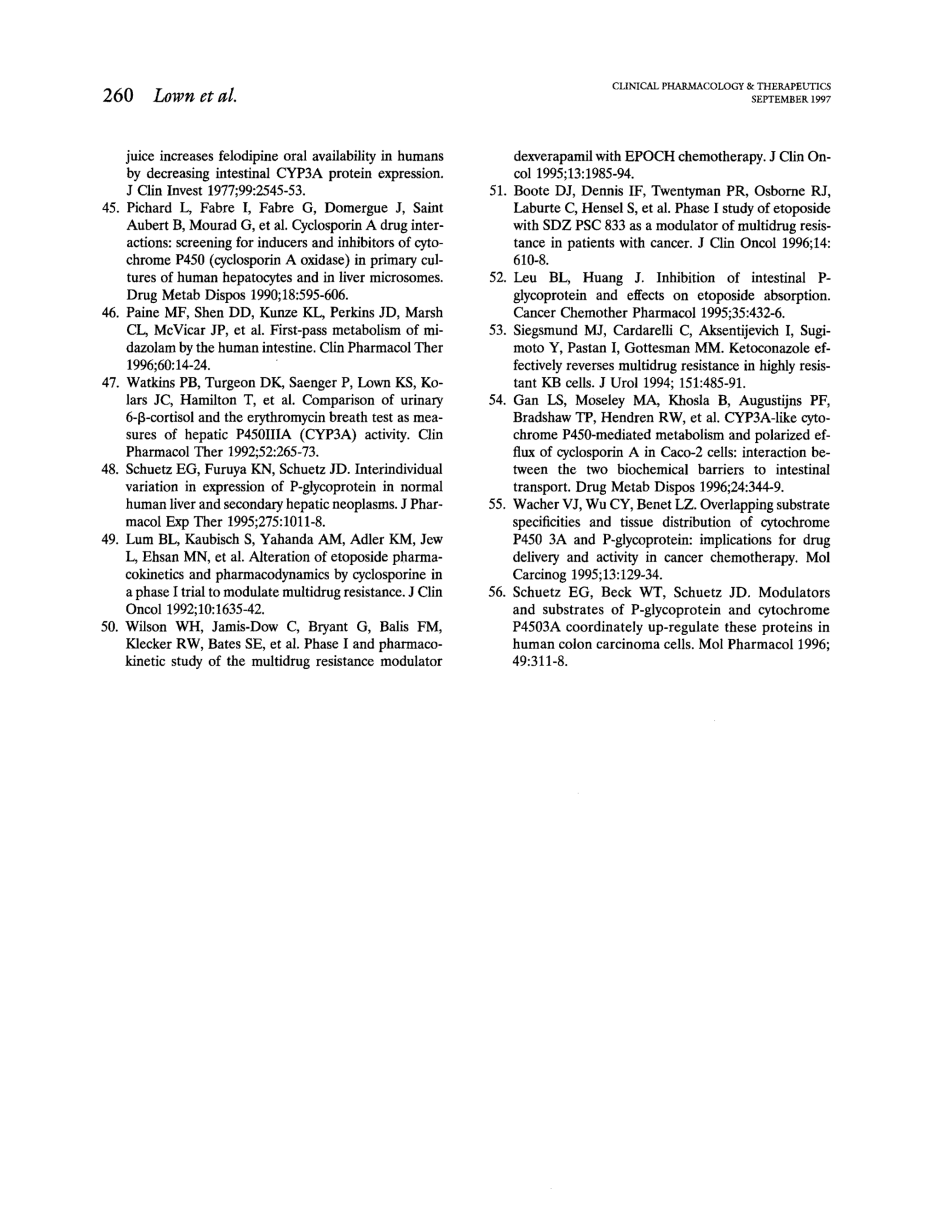# Deciphering the Relative Contribution of CYP3A4 Versus P‐Glycoprotein for the Shared Substrate Cyclosporine—Commentary on Lown *et al*.

**DOI:** 10.1002/cpt.3619

**Published:** 2025-05-19

**Authors:** Ingolf Cascorbi, Richard B. Kim

**Affiliations:** ^1^ Institute of Experimental and Clinical Pharmacology University Hospital Schleswig‐Holstein Kiel Germany; ^2^ Division of Clinical Pharmacology Western University London Ontario Canada

## Abstract

The oral bioavailability of cyclosporine, a substrate of both CYP3A4 and P‐glycoprotein, is subject to large inter‐individual variability, which requires frequent monitoring of plasma concentrations. In 1997, the study by Lown *et al*. showed that—in addition to hepatic CYP3A4—the expression of P‐gp in the intestine significantly influences the pharmacokinetics of cyclosporine in kidney transplant patients. The results contributed considerably to a better understanding of the function of the intestinal P‐glycoprotein for drug clearance.

Today it is well established that the drug biodistribution and disposition profiles are closely linked to the expression and activity of absorption, distribution, metabolism, and excretion (ADME) genes. For many drugs in clinical use, in addition to drug metabolizing enzymes such as cytochrome P450s, drug transporters that mediate the cellular uptake as well as efflux have also been established as major determinants of drug disposition and response.^1^ Among the drug transporters studied to date, there is little doubt that P‐glycoprotein (gene name *ABCB1*) is one of the best‐characterized human drug transporters. P‐glycoprotein (P‐gp) is widely appreciated for its broad substrate specificity as well as its expression on the luminal or apical cell membrane domain of intestinal enterocytes, hepatocytes, endothelial cells of brain capillaries, tubular cells of the kidneys and in a number of other organs in the body, and functions to limit tissue drug entry or enhances elimination of substrate drugs from the body.^2^

Our knowledge regarding P‐gp substrate specificity, expression, and function, including its role in chemotherapy drug resistance and drug disposition had developed over many decades.^3^, ^4^ The 1990s represented a decade in which a number of pivotal studies on the role of P‐gp as a potential determinant of inter‐subject variation in the pharmacokinetic profile of drugs were carried out. During this period, studies using P‐gp knockout mice provided compelling evidence for P‐gp as a major player in drug clearance and its broad substrate specificity often overlapped with the drug‐metabolizing enzyme, CYP3A4.^5^, ^6^ The putative potential for P‐gp as a contributor to variation in drug disposition was supported by the observation that the expression of P‐gp was highly variable in liver samples from healthy individuals as well as those with secondary hepatic neoplasms.^7^

In a study by Thummel *et al*. the immunosuppressant cyclosporine, a known P‐gp substrate, showed that varying CYP3A4 content accounted for two third of interpatient variation when cyclosporine was administered intravenously, but CYP3A4 expression could not explain the variability of cyclosporine pharmacokinetics when the drug was given orally.^8^, ^9^

A clinical study focusing on cyclosporine pharmacokinetics, from the laboratory of Paul Watkins, who at the time was in Ann Arbor, MI in collaboration with Leslie Benet in San Francisco, CA, led to a ground‐breaking finding on the clinical relevance of P‐gp, particularly at the level of the intestine, to the observed pharmacokinetic profile of cyclosporine.^10^ This paper turned out to be the highest cited original research article published in *Clinical Pharmacology and Therapeutics* during the decade 1990–1999.

In this elegant study of 25 patients with kidney transplantation at steady‐state during oral cyclosporine therapy, the correlation of plasma PK of cyclosporine with hepatic CYP3A4 activity and duodenal CYP3A4 and P‐gp expression was analyzed. After the exclusion of patients whose 0 and 24 h cyclosporine blood concentrations differed by more than 25% (considered to be not at steady‐state) and of one patient taking the CYP3A4/P‐gp inducer phenytoin, there was still a four to sevenfold variability of plasma AUC, *C*_max_, *t*_max,_ and CL/F, but log CL/F correlated significantly with erythromycin breath that reflected hepatic CYP3A4 activity. A stepwise forward multiple regression analysis however revealed that also intestinal P‐gp expression (determined from duodenal biopsies by endoscopies) contributed to 17% of variation thereby improving the CL/F‐prediction of the model from *r* ^2^ = 0.56 to 0.73. Interestingly, other factors including intestinal CYP3A4 did not further improve the model. Of note, there was no correlation between intestinal P‐gp abundance and intestinal or hepatic CYP3A4 expression or activity, respectively explaining also that there was no direct correlation between intestinal P‐gp expression with cyclosporine clearance. It was concluded that both, hepatic CYP3A4 and intestinal P‐gp expression are key determinants of interindividual variability of cyclosporine pharmacokinetics and that hepatic CYP3A4 activity was highly variable. In contrast, cyclosporine *c*_max_ variation could be explained to 62% by intestinal P‐gp, but only to 32% with results from the erythromycin breath test.

Overall, this study contributed substantially to our understanding of P‐gp as an efflux pump limiting the uptake of a number of drugs not only in the liver, but also at the level of the intestine. It also helped to explain the earlier studies on drug–drug interactions of renally excreted drugs like digoxin with the inducer rifampicin^11^ and also for the more recently approved oral anticoagulant such dabigatran‐etexilate, a prodrug of dabigatran. Interestingly, only the etexilate form is a P‐gp‐substrate, but not dabigatran itself, hence interactions take place at the level of the intestine.^12^ Moreover, the observations also underscore the problem of substantial overlaps of substrates and inhibitors, for example, CYP3A4 and P‐gp that have to be considered in drug–drug interaction studies.^13^ For instance, both rifampicin and St. John's wort may induce CYP3A and P‐gp leading to a substantial decrease in cyclosporine plasma concentrations thereby increasing the risk of transplant rejection.^14^, ^15^

It should be noted that the observations on the variability of intestinal P‐gp‐expression were also the starting point of systematic sequencing of the *ABCB1* gene.^16^ After conflicting studies on the role of *ABCB1* genetic variation on the bioavailability of cyclosporine and its effects,^17^, ^18^, ^19^ currently, it does not appear common *ABCB1* single nucleotide variations or haplotypes predict individual drug dosing in the setting of organ transplantation drug therapy.^20^ Although a lot of efforts have been made to investigate the functional consequences of *ABCB1* genetic variation on cellular and clinical levels, with respect to the observations of Lown *et al*.,^10^ our current knowledge of *ABCB1* pharmacogenetics does not adequately explain the observed large interindividual variation of P‐gp expression.

In conclusion, the highlighted study by Lown *et al*. demonstrates the impact of a thoughtfully designed clinical study for a better understanding of the role of ADME genes in different compartments and tissue barriers where the resultant findings could then lead to a more accurate/predictive physiologically based pharmacokinetic/pharmacodynamic model during the drug development process.

## FUNDING

No funding was received for this work.

## CONFLICT OF INTEREST

The authors declared no competing interests for this work. As Associate Editors of *Clinical Pharmacology & Therapeutics*, Ingolf Cascorbi and Richard Kim were not involved in the review or decision process for this paper.